# Genome-wide association studies using multi-models and multi-SNP datasets provide new insights into pasmo resistance in flax

**DOI:** 10.3389/fpls.2023.1229457

**Published:** 2023-10-25

**Authors:** Liqiang He, Yao Sui, Yanru Che, Huixian Wang, Khalid Y. Rashid, Sylvie Cloutier, Frank M. You

**Affiliations:** ^1^Ottawa Research and Development Centre, Agriculture and Agri-Food Canada, Ottawa, ON, Canada; ^2^School of Tropical Agriculture and Forestry, School of Tropical Crops, Hainan University, Haikou, China

**Keywords:** GWAS, multi-locus model, pasmo, SNP, flax

## Abstract

**Introduction:**

Flax (*Linum usitatissimum* L.) is an economically important crop due to its oil and fiber. However, it is prone to various diseases, including pasmo caused by the fungus *Septoria linicola*.

**Methods:**

In this study, we conducted field evaluations of 445 flax accessions over a five-year period (2012–2016) to assess their resistance to pasmo A total of 246,035 single nucleotide polymorphisms (SNPs) were used for genetic analysis. Four statistical models, including the single-locus model GEMMA and the multi-locus models FarmCPU, mrMLM, and 3VmrMLM, were assessed to identify quantitative trait nucleotides (QTNs) associated with pasmo resistance.

**Results:**

We identified 372 significant QTNs or 132 tag QTNs associated with pasmo resistance from five pasmo resistance datasets (PAS2012–PAS2016 and the 5-year average, namely PASmean) and three genotypic datasets (the all SNPs/ALL, the gene-based SNPs/GB and the RGA-based SNPs/RGAB). The tag QTNs had *R*^2^ values of 0.66–16.98% from the ALL SNP dataset, 0.68–20.54%from the GB SNP dataset, and 0.52–22.42% from the RGAB SNP dataset. Of these tag QTNs, 93 were novel. Additionally, 37 resistance gene analogs (RGAs)co-localizing with 39 tag QTNs were considered as potential candidates for controlling pasmo resistance in flax and 50 QTN-by-environment interactions(QEIs) were identified to account for genes by environmental interactions. Nine RGAs were predicted as candidate genes for ten QEIs.

**Discussion:**

Our results suggest that pasmo resistance in flax is polygenic and potentially influenced by environmental factors. The identified QTNs provide potential targets for improving pasmo resistance in flax breeding programs. This study sheds light on the genetic basis of pasmo resistance and highlights the importance of considering both genetic and environmental factors in breeding programs for flax.

## Introduction

Flax (*Linum usitatissimum* L.) is a valuable economic crop that provides linseed and stem fiber to humans (Singh et al., 2011; You et al., 2017). However, flax production is often constrained by pasmo, a disease caused by the fungus *Septoria linicola*, which reduces seed yield and fiber quality (Halley et al., 2004; He et al., 2018; Islam et al., 2021). The fungus infects flax from the seedling to the ripening stages. At the flowering stage, despite the application of fungicide, susceptible varieties have been reported to experience up to a 75% seed yield loss (Hall et al., 2016; Islam et al., 2021). Therefore, developing resistant varieties is a cost-effective and environmentally-friendly approach to protect flax from pasmo and its effects on yield.

Disease resistance in plants is typically quantitatively inherited and influenced by the environment. It is primarily governed by major resistant genes called *R* genes, which have been the topic of many studies (Marone et al., 2013; Yang et al., 2017). Most cloned *R* genes in plants belong to the nucleotide-binding site-leucine-rich repeat domain (NBS-LRR) class, also known as *NLRs*. For example, a cluster of *NLR* receptor-encoding genes confers durable resistance to *Magnaporthe oryzae* in rice (Deng et al., 2017), and the *rp1* gene in maize and its homolog in barley confer race-specific resistance to rust fungal diseases (Collins et al., 1999; Ayliffe et al., 2000). *Receptor like kinase* (*RLK*) genes also account for a significant proportion of *R* genes. For instance, the RLK-encoding barley *Rpg1* gene confers resistance to stem rust (Brueggeman et al., 2002), and rice *Pi-d2* gene confers resistance against rice blast (Chen et al., 2006). Transmembrane coiled-coil proteins (TM-CC) are another essential type of *R* gene-encoded proteins. The *Rph3* gene, originating from wild barley, is a TM-type *R* gene that encodes a protein that differs from all known plant disease resistance proteins and can significantly enhance barley leaf rust resistance (Dinh et al., 2022). The mutation-induced recessive *mlo* allele of the barley *Mlo* gene also encodes a TM domain protein, and confers broad-spectrum resistance to the fungal pathogen *Erysiphe graminis* (Buschges et al., 1997). Resistance gene analogs (RGAs) are key resistance gene candidates and have been well-characterized in flax (Sekhwal et al., 2015; You et al., 2018b). A total of 1327 RGAs have been categorized into 11 types: RLK (receptor-like protein kinase), TM-CC (transmembrane coiled-coil protein), RLP (receptor-like protein), TNL (TIR-NBS-LRRs), TX (TIR-unknown), NL (NBS-LRR), CNL (CC-NBS-LRR), TN (TIR-NBS), NBS (NBS domain only), CN (CC-NBS), and OTHERS.

Genome-wide association studies (GWAS) have emerged as a powerful and efficient approach for unraveling the genetic basis of complex traits in flax. Compared to traditional linkage mapping, GWAS can achieve higher resolution and more accurate mapping of quantitative trait nucleotides (QTNs) (He et al., 2018; You et al., 2018a; Soto-Cerda et al., 2021; You et al., 2022). However, GWAS has some limitations, including a higher risk of false-positive associations and a lower effectiveness in detecting quantitative trait loci (QTL) associated with rare alleles than biparental populations. Single-locus GWAS models, such as GEMMA and MLM, have proven to be effective in controlling spurious associations using the stringent Bonferroni correction but they are not suited to detecting minor QTL (Yu et al., 2006; Zhou and Stephens, 2012). To enhance the power of polygenic loci detection, multi-locus GWAS models have been developed (Segura et al., 2012; Zhang et al., 2019b). For instance, FarmCPU improves statistical power and reduces confounding associations (Liu et al., 2016), and mrMLM increases power, reduces the false positive rate, and has a shorter running time (Wang et al., 2016). However, these models do not fully assess the effects of QTN-by-environment interactions (QEIs) and QTN-by-QTN interactions (QQIs). To address these, a new multi-locus GWAS model called 3VmrMLM was proposed (Li et al., 2022b). This model estimates the genetic effects of three marker genotypes (AA, Aa and aa) while controlling all possible polygenic backgrounds. It is designed to detect QEIs and QQIs. Our previous study has shown that pasmo resistance in flax is controlled by polygenes (He et al., 2018). However, the small proportion of resistant accessions in the original core collection was limiting and additional research is warranted to detect main-effect QTNs and their corresponding causal genes. Furthermore, the QEIs associated with flax pasmo resistance are still largely unknown. Therefore, the newly released 3VmrMLM model to identify main-effect QTNs and QEIs is expected to improve our understanding of pasmo resistance in flax towards the better design of breeding solutions.

Our previous study has identified a total of 500 QTL associated with pasmo resistance in flax, including 67 stable and large-effect QTL and many additional small effect and environment-specific QTL (He et al., 2018). Here only 8.3% of the flax core collection was found to be resistant or moderately resistant to pasmo, based on the average pasmo severity over five consecutive years (2012–2016). To increase the proportion of resistant lines in the collection while simultaneously improving genetic diversity, 75 sequenced breeding lines were added to the core collection. Pasmo resistance data for these new lines, were collected between 2012 and 2016, alongside data from the existing 370 original accessions of the flax core collection (You et al., 2022; Zheng et al., 2023).

To gain a deeper understanding of pasmo resistance in flax at the genetic level, we conducted a GWAS on a diverse panel of 445 flax accessions, which included 370 accessions of the core collection and 75 selected breeding lines (SBLs). Compared to GWAS that use all SNPs (ALL) as genotypic data, gene-based SNPs (GB) and RGA-based SNPs (RGAB) GWAS have demonstrated higher power and resolution in QTL detection and candidate gene identification (Zhang et al., 2021; You et al., 2022). Thus, three genotypic datasets consisting of 246,035 SNPs (ALL), 65,147 SNPs within genes (GB), and 3,510 SNPs within RGAs (RGAB) were used in the analysis, along with four different GWAS models. These models included one single-locus model (GEMMA) and three multi-locus models (FarmCPU, mrMLM, and 3VmrMLM), employed to detect quantitative trait nucleotides (QTNs) and QTN-by-environment interactions (QEIs) associated with pasmo resistance across five individual years (2012–2016). Our goal was to identify potential candidate genes conferring pasmo resistance in flax.

## Materials and methods

### Genetic panel for GWAS

**Figure 1:**
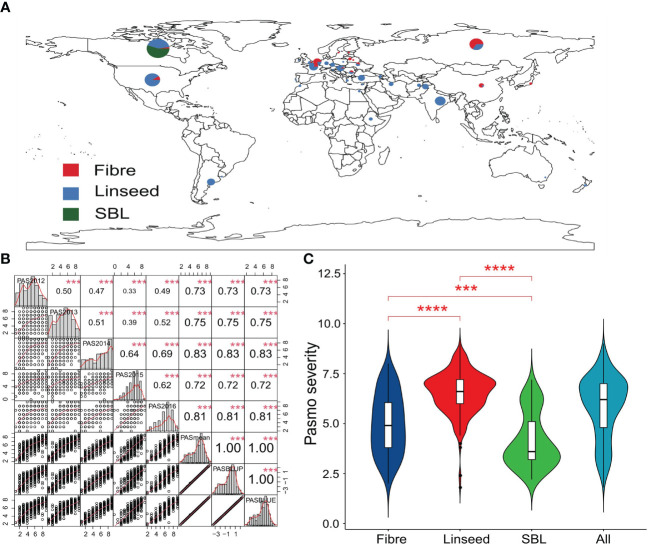
Geographic distribution and phenotyping for pasmo resistance in flax accessions. **(A)** Geographic distribution of 445 flax accessions. **(B)** Distribution and correlation matrix of pasmo severity in five consecutive years (2012–2016), mean, BLUP and BLUE pasmo severity over years. *** indicates significant correlation at the 0.1% probability level. **(C)** Violin plot of pasmo severity for the 80 fibre and 290 linseed accessions of the core collection and the 75 selected breeding lines. PAS2012, PAS2013, PAS2014, PAS2015, PAS2016, PASmean, PASBLUP and PASBLUE represent pasmo severity datasets for 2012, 2013, 2014, 2015, 2016, the 5-year average, the best linear unbiased prediction values and the best linear unbiased estimation values of pasmo severity over five years. *** and **** indicate statistical significance at the 0.1% and 0.01% probability level, respectively.

A genetic panel of 445 flax accessions was used for GWAS. The panel included 370 accessions from the flax core collection, which was previously assembled from a worldwide collection of 3,378 flax accessions (Diederichsen et al., 2012; Soto-Cerda et al., 2013; He et al., 2018), and 75 breeding lines that were selected based on their resistance to pasmo, *Fusarium* wilt and powdery mildew diseases (You et al., 2022). The flax core collection included accessions from 11 geographical origins, and were classified based on their morphotype into 80 fibre and 290 linseed accessions. This panel included 17 landraces, 85 breeding lines, 232 cultivars, and 36 accessions of unknown improvement status (**Figure 1A**) (You et al., 2017). By adding the 75 SBLs to the core collection, the statistical power of the GWAS was increased. This diverse genetic panel allows for a more comprehensive analysis of the genetic variation within flax, and can provide insights into the genetic basis of resistance to pasmo disease and other traits of interest.

### Phenotyping of pasmo resistance and statistical analysis

The 445 accessions of the diversity panel were evaluated for field resistance to pasmo over a period of five years (2012–2016) at Agriculture and Agri-Food Canada, Morden Research and Development Center’s farm in Morden, Manitoba, Canada. A Type-2 modified augmented design (MAD2) was employed for the field experiments as described by You et al. (2017). The seeds were sown in mid-May each year, and 30-centimeter tall flax plants were inoculated with approximately 200 grams of pasmo-infected chopped straw from the previous growing season. To ensure disease infection and development, a spray system was operated for 5 minutes every half hour for 4 weeks.

Pasmo resistance was evaluated at the early brown boll stage (21–30 days after the flowering) by assessing the leaves and stems of all plants (~300) in a single row plot using a pasmo severity scale of 0–9. Ratings of 0–2 were classified as resistant (R), 3–4 as moderately resistant (MR), 5–6 as moderately susceptible (MS), and 7–9 as susceptible (S). Pasmo severity data were recorded for five individual years (PAS2012, PAS2013, PAS2014, PAS2015, and PAS2016). These five datasets and the five-year average (PASmean) were used as the phenotypic data for all analyses in this study.

To account for environmental variation, the R package lme4 was used to generate the best linear unbiased prediction (BLUP) and best linear unbiased estimate (BLUE) datasets for the pasmo severity of the five years (Bates et al., 2015). A mixed linear model that treated accessions and years as random effects was used to calculate the BLUP values, while another mixed linear model that treated accessions as fixed effects and years as random effects was employed to obtain the BLUE values. The R package PerformanceAnalytics was used to analyze the correlations between the pasmo severity datasets, and to generate histograms and scatter plots (https://cran.r-project.org/web/packages/PerformanceAnalytics/index.html).

### Re-sequencing for SNP discovery of the diversity panel

Genome re-sequencing was performed to obtain the genetic variation of 445 flax accessions. As previously described in He et al. (2018), the Illumina HiSeq 2000 platform (Illumina Inc., San Diego, USA) was used to generate 100-bp paired-end reads with an average coverage of ~15.5X of the reference genome. All raw reads were mapped to the flax reference genome using the BWA v0.6.1 mapping tool with a base-quality Q score in Phred scale > 20 and other default parameters (Jo and Koh, 2015). The mapped files were processed using SAMtools and an improved AGSNP pipeline for SNP calling (Li et al., 2009; You et al., 2011; You et al., 2012). The detected SNPs were further filtered with a minor allele frequency (MAF) > 0.05 and a SNP genotyping call rate ≥ 60% using PLINK (https://zzz.bwh.harvard.edu/plink/). After linkage disequilibrium (LD) filtering with pairwise correlation coefficients (*r^2^*) among neighboring SNPs within 200kb > 0.8 and Beagle imputation with default parameters (Browning and Browning, 2007), a total of 246,035 high-quality SNPs were retained for further analysis. The genetic variant annotation and functional effect prediction of each SNP were characterized by snpEff software (Cingolani et al., 2012) based on the reference genome and corresponding annotation (You et al., 2018b).

### Population structure analysis

To dissect the genetic structure and variation of the 445 flax accessions, principal component analysis (PCA) was performed using the obtained high-quality SNPs. The analysis was carried out with the PLINK software (Elhaik, 2022). For the SNP-based phylogenetic analysis, MEGA-CC was employed, using a pairwise gap deletion method for 1,000 bootstrap replicates (Kumar et al., 2012). The resulting phylogenetic tree was visualized using the Interactive Tree of Life (iTOL) tool (Letunic and Bork, 2021). The population stratification was estimated using ADMIXTURE (Alexander et al., 2009). The genome-wide LD decay was assessed using PopLDdecay v3.42 software to the squared correlation coefficient (*r*^2^) between SNPs (Zhang et al., 2019a).

### Genome-wide association study

The GWAS analysis for pasmo resistance was conducted using the five individual year (PAS2012, PAS2013, PAS2014, PAS2015, and PAS2016) and the five-year average (PASmean) datasets with four GWAS models. The models used included the single-locus model GEMMA and the multi-locus models FarmCPU (Liu et al., 2016), mrMLM (Wang et al., 2016) and 3VmrMLM (Li et al., 2022b). The kinship matrices were estimated using the protocol suggested by each GWAS software package. The genotypic data for the association panel comprised 246,035 high-quality SNPs (ALL) obtained from 445 flax accessions. Of these, the 65,147 SNPs that mapped to the genic regions constituted the gene-based (GB) SNP dataset, and the 3,510 SNPs that mapped to RGAs formed the RGA-based (RGAB) SNP dataset. These datasets were used in sequential analyses. The GEMMA software and R package GAPIT were employed to detect QTNs using default settings (Zhou and Stephens, 2012; Wang and Zhang, 2021). The R package mrMLM was applied to detect QTNs using parameters SearchRadius = 20, CriLOD = 3, and Bootstrap = FALSE (Zhang et al., 2020). The R package IIIVmrMLM implementing the 3VmrMLM model was used to detect main-effect QTNs and the QEIs (Li et al., 2022a). For the detection of the main-effect QTNs, the R package IIIVmrMLM was used with the following parameters: method = “Single_env”, SearchRadius = 20, and svpal = 0.01. For QEI detection, the parameters used were method = “Multi_env”, SearchRadius = 20, and svpal = 0.01. The association signals of the 3VmrMLM model were detected using a LOD score ≥ 3 (Li et al., 2022a). The threshold of significant association of GEMMA and FarmCPU was determined using a critical *P*-value at the 5% significant level that was subjected to Bonferroni correction (*P*-value = 2.03 × 10^−7^ for the ALL dataset, *P*-value = 7.67 × 10*^−^*^7^ for the GB dataset, and *P*-value = 1.42 × 10^−5^ for the RGAB dataset). Manhattan plots were generated using the IIIVmrMLM package with default settings.

### QTN identification, candidate gene prediction, allele and haplotype analysis

In order to identify QTNs associated with pasmo resistance in flax, a GWAS was performed using individual year datasets (PAS2012–PAS2016) and a five-year average dataset (PASmean) in combination with the ALL, GB and RGAB genotypic datasets. QTNs detected in different genotypic datasets were analyzed independently and common QTNs were identified based on detection by two or more models or detection in two or more phenotypic datasets. Mann-Whitney U tests were used to validate significant differences between QTN alleles associated with pasmo severity. The significant QTNs were represented by tag QTNs for downstream analyses. *R^2^* values were calculated to determine the proportion of total variation explained by the pasmo resistance associated QTNs/QEIs. A total of 1,327 RGAs have previously been identified in the flax reference genome (You et al., 2018b). The co-localized RGAs within an estimated 4 kb distance of the averaged whole genome LD decay and local LD block defined flanking regions of the detected QTNs/QEIs were considered as candidate genes. LDBlockShow v1.40 (Dong et al., 2021) was utilized to estimate the local LD block regions on the chromosomes. For allele analysis, the single SNP with HIGH functional effect prediction on the coding region (CDS) of each candidate gene were selected and tested for significant differences in pasmo severity using the Wilcox non-parametric test at the 5% probability level. Likewise, for haplotype analysis, all the SNPs within each candidate gene that were predicted with HIGH or MODERATE functional effect were considered. Subsequently, these SNPs underwent testing using the Wilcox non-parametric test at the 5% probability level to identify significant differences. A SNP with a HIGH functional effect prediction is assumed to have a disruptive impact on the protein, while a SNP with a MODERATE functional effect prediction is expected to be non-disruptive but could possibly change the protein’s effectiveness.

## Results

### Evaluation of pasmo resistance

Pasmo resistance was evaluated in 445 flax accessions over five consecutive years (PAS2012–PAS2016). The geographic distribution and morphotypes of these accessions are shown in **Figure 1A**. Correlation coefficients were calculated among PAS2012, PAS2013, PAS2014, PAS2015, PAS2016, PASmean, pasmo best linear unbiased prediction (PASBLUP) and pasmo best linear unbiased estimation (PASBLUE) datasets, and ranged from 0.33 to 1.00, with the highest correlation observed between PASmean and PAS2014 (*r* = 0.83) (**Figure 1B**). PASmean was further analyzed due to its almost identical correlation coefficients with PASBLUP and PASBLUE (*r* = 1.00). The coefficient of variation (CV) of PAS2012–PAS2016 and PASmean datasets ranged from 24.17% to 39.24% (**Supplementary Table S1**). Significant differences in pasmo severity were observed between linseed, fibre accessions, and SBLs in this flax genetic panel. High resistance (low severity) to pasmo was observed in the 75 SBLs compared to the 370 accessions from the flax core collection (**Figure 1C**). The average pasmo severity over five years was 6.56 ± 1.05 for the 290 linseed accessions, 4.98 ± 1.50 for the 80 fibre accessions, and 4.13 ± 1.35 for the 75 breeding lines (**Figure 1C**). The data distribution and correlation analysis indicated that resistance against pasmo in flax is controlled by polygenes and potentially genetic by environment interactions.

### Population structure

**Figure 2:**
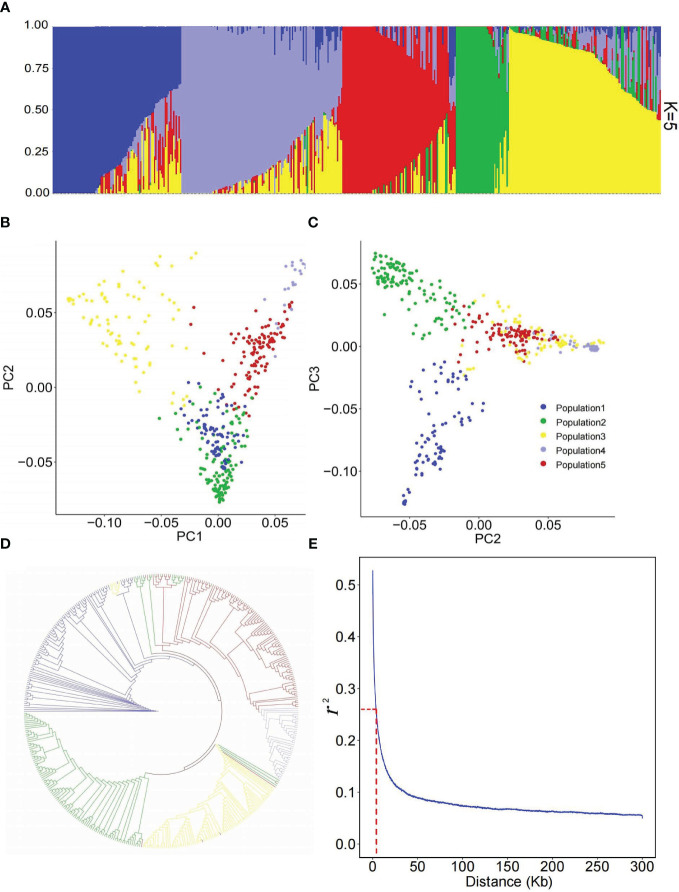
Population structure of 445 flax accessions. **(A)** Population structure estimated by ADMIXTURE. **(B, C)** Scatter plots of the first three principal components (PCs) of 445 flax accessions. **(D)** Phylogenetic analysis of 445 flax accessions based on 246,035 single nucleotide polymorphisms (SNPs). Accessions of clades one, two, three, four and five are indicated in blue, green, yellow, mauve and red, respectively. **(E)** Genome-wide LD decay analysis of the genetic panel.

To analyze the genetic structure of the 445 flax accessions, a population structure analysis was performed using the ALL SNP dataset of 246,035 SNPs. The results indicated the 445 accessions were divided into five populations (**Figure 2A**). Population one consisted of 19 linseed accessions and 75 SBLs; population two was composed of 67 fibre accessions and 51 linseed accessions; population three contained 11 fibre accessions and 72 linseed accessions; population four comprised 39 linseed accessions, while population five consisted of only two fibre accessions and 109 linseed accessions. PCA and phylogenetic analysis by neighbor-joining (NJ) (Chen et al., 2014) also showed identical classification of the flax genetic panel into five groups (**Figures 2B–D** and **Supplementary Figure S1**). Therefore, a population structure Q matrix with K = 5 was adopted for downstream GWAS analyses. The linkage disequilibrium (LD) analysis showed that the LD decayed rapidly before 4 kb and subsequently became flat for this flax genetic panel (**Figure 2E**). Therefore, the 4 kb flanking region of each QTN was used for putative candidate gene prediction in subsequent analyses.

### Identification of QTNs associated with pasmo resistance

**Figure 3:**
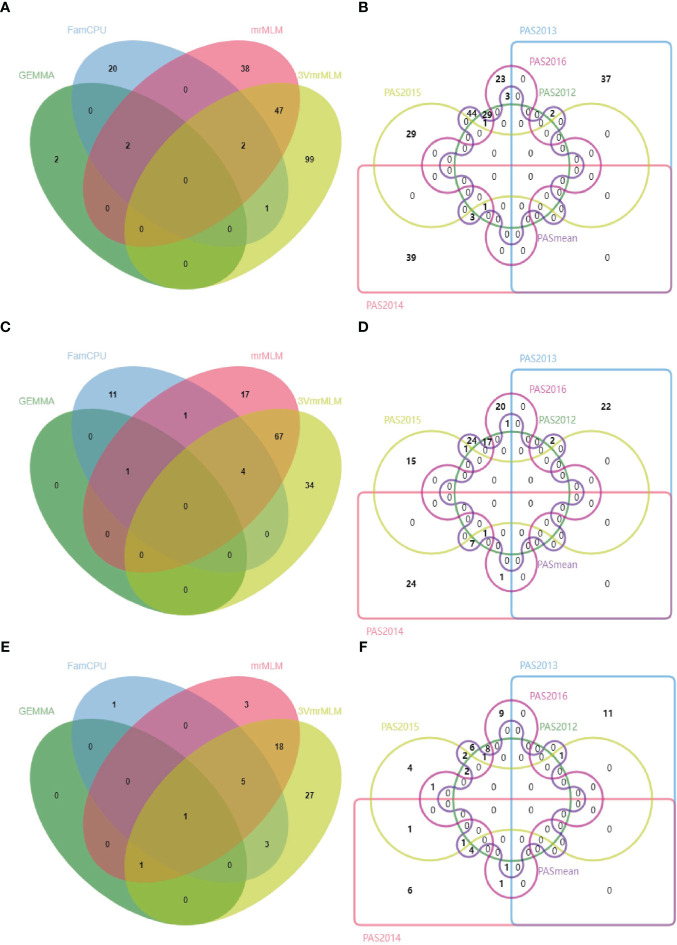
Venn diagrams of QTNs detected using four GWAS models (GEMMA, FarmCPU, mrMLM, and 3VmrMLM) for the three single nucleotide polymorphism (SNP) datasets: ALL **(A)**, GB **(C)**, and RGAB **(E)**, and QTNs detected using six different phenotypic datasets (PAS2012–PAS2016 and PASmean) for the three SNP datasets: ALL **(B)**, GB **(D)**, and RGAB **(F)**. ALL, all SNPs; GB, gene-based SNPs; RGAB, resistance gene analog (RGA) -based SNPs.

A total of 372 significant QTNs were identified using six pasmo resistance datasets (PAS2012–PAS2016 and PASmean) and three genotypic datasets (ALL, GB and RGAB) using the single-locus model GEMMA and the multi-locus models FarmCPU, mrMLM and 3VmrMLM (**Figure 3** and **Supplementary Table S2**). When the ALL genotypic dataset was used, 3VmrMLM detected the most QTNs (149), followed by mrMLM (89), FarmCPU (25), and GEMMA (4) (**Table 1**). Forty-seven QTNs were detected by both 3VmrMLM and mrMLM, two by 3VmrMLM, mrMLM, and FarmCPU, and another two by mrMLM, FarmCPU, and GEMMA (**Figure 3A**). Only one QTN (QTN-Lu4-14738243) was detected in three out of the six phenotypic datasets (PAS2012–PAS2016 and PASmean) (**Figure 3B** and **Supplementary Table S2**).

**Table 1:** Comparison of quantitative trait nucleotide (QTN) identification for different GWAS models and genotypic datasets.

Statistical model	Genotypic dataset	NO. of detected QTNs	NO. of common QTNs by models or datasets	NO. of non–significant QTNs	NO. of tag QTNs	*R^2^* range (%)
GEMMA	ALL	4	2	0	2	1.11–10.00
FarmCPU	ALL	25	6	0	6	1.11–12.11
mrMLM	ALL	89	51	10	41	0.66–12.72
3VmrMLM	ALL	149	52	12	41	0.66–16.98
GEMMA	GB	1	1	0	1	1.11
FarmCPU	GB	17	8	1	7	1.11–13.30
mrMLM	GB	90	74	12	62	0.68–20.54
3VmrMLM	GB	105	75	13	62	0.68–20.54
GEMMA	RGAB	2	2	0	2	9.34–22.42
FarmCPU	RGAB	10	9	2	7	0.54–22.42
mrMLM	RGAB	28	25	4	23	0.52–17.40
3VmrMLM	RGAB	55	32	3	30	0.52–17.40

ALL, all SNPs: GB, gene SNPs: RGAB, resistance gene analog (RGA) based SNPs.

For the GB genotypic dataset, 3VmrMLM detected the most QTNs (105), followed by mrMLM (90), and GEMMA detected a single QTN (**Table 1**). Among these, 67 were detected by both 3VmrMLM and mrMLM, four by 3VmrMLM, mrMLM, and FarmCPU, and one by mrMLM, FarmCPU, and GEMMA (**Figure 3C**). Moreover, the same common QTN (QTN-Lu4-14738243) was detected in three out of the six phenotypic datasets (**Figure 3D** and **Supplementary Table S2**).

Similarly, 3VmrMLM detected the most QTNs (55) in the RGAB genotypic dataset, followed by mrMLM (28), FarmCPU (10), and GEMMA (2) (**Table 1**). Interestingly, QTN-Lu10-11656889 was detected by all four models (**Figure 3E** and **Supplementary Table S2**). Besides, three common QTNs (QTN-Lu8-23634276, QTN-Lu10-11656889, and QTN-Lu15-14719354) were detected in three out of six phenotypic datasets (**Figure 3F** and **Supplementary Table S2**). Notably, QTN-Lu14-2333894 was detected by all three genotypic datasets (**Supplementary Figure S2A** and **Supplementary Table S2**).

In summary, 3VmrMLM detected the highest number of total QTNs and common QTNs in the six phenotypic datasets regardless of the genotypic dataset. The largest number of QTNs detected in multiple environments (three out of six phenotypic datasets) was identified using the RGAB genotypic dataset.

**Figure 4:**
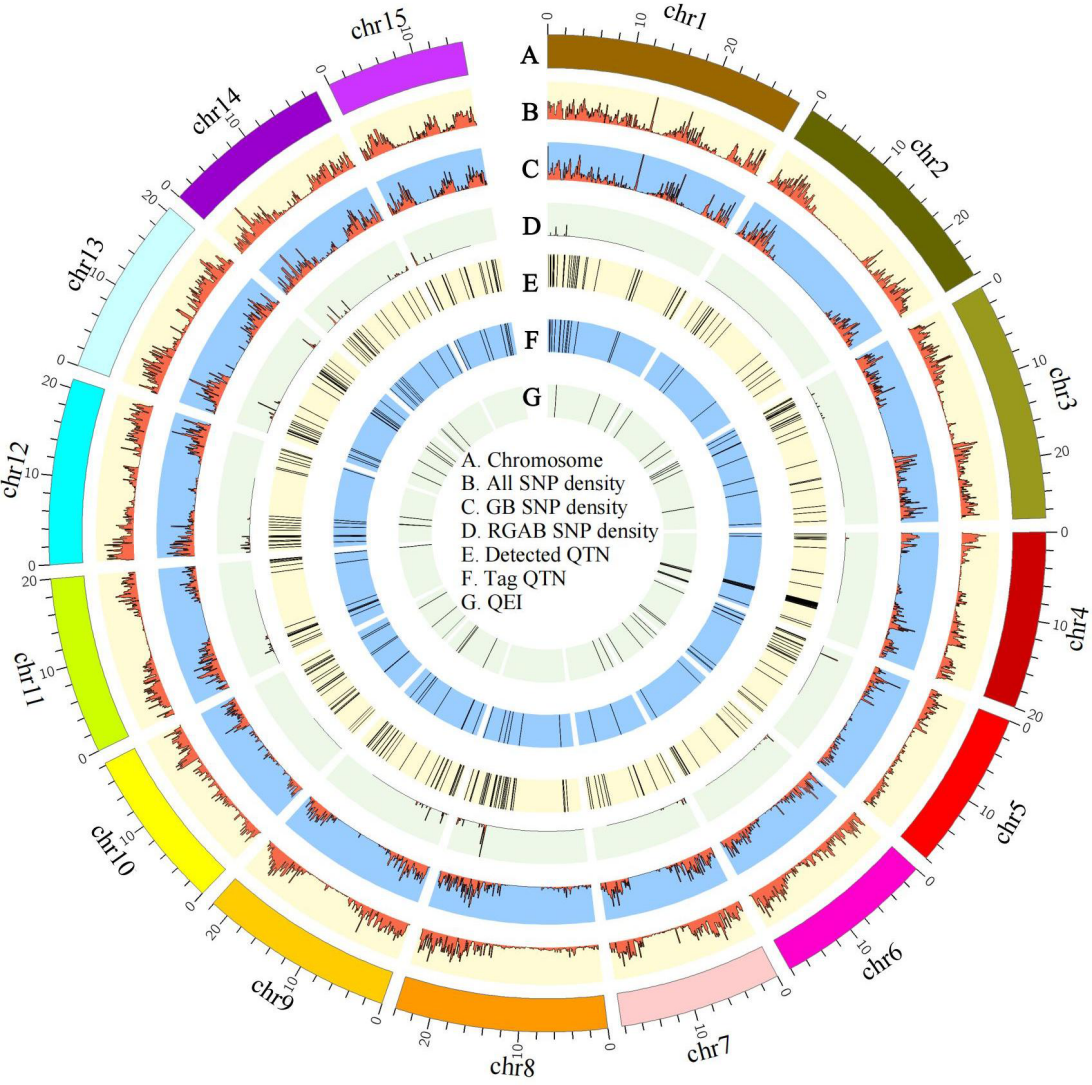
Circos map of quantitative trait nucleotides (QTNs) and QTN-by-environment interactions (QEIs) for pasmo severity in flax. Track A: 15 flax chromosomes. Track B: Heatmap of SNP density with bin sizes of 0.1 Mb for the ALL dataset (246,035 SNPs). Track C: Heatmap of SNP density with bin size of 0.1 Mb for the GB dataset (65,147 SNPs). Track D: Heatmap of SNP density with bin size of 0.1 Mb for the RGAB dataset (3,510 SNPs). Track E: QTNs detected using four statistical models: GEMMA, FarmCPU, mrMLM, and 3VmrMLM. Track F: QTNs identified using all four statistical models. Track G: QEIs detected using the 3VmrMLM model. ALL, all SNPs; GB, gene-based SNPs; RGAB, resistance gene analog (RGA)-based SNPs.

All significant QTNs were evaluated for consistency across multiple phenotypic datasets and models, and those detected in ≥ two datasets or ≥ two models were retained for further analysis. A total of 55, 80, and 32 QTNs were thus identified from the ALL, GB, and RGAB genotypic datasets, respectively (**Supplementary Table S2**). In agreement with the total number of QTNs detected, the majority of the retained QTNs were detected by 3VmrMLM across all three genotypic datasets, with 52 QTNs in ALL, 75 QTNs in GB, and 32 QTNs in RGAB (**Table 1** and **Supplementary Table S2**). Allelic test of significance for these QTNs were performed using the Mann-Whitney U test for the dataset from which the QTNs were detected. A total of 82 non-significant QTNs (U test at the 5% probability level) were removed, leaving 132 significant QTNs used as tag QTNs in subsequent analyses (**Figure 4** and **Supplementary Tables S2**, **S3**). The majority of the tag QTNs were detected by 3VmrMLM across all three genotypic datasets, with 41 in ALL, 62 in GB, and 30 in RGAB (**Table 1**). The *R*^2^ values of the 132 tag QTNs ranged from 0.52% to 22.42% (**Table 1** and **Supplementary Table S3**), and varied across the four models due to the differences in statistical models. For example, the *R*^2^ of 3VmrMLM-detected tag QTNs in the ALL genotypic dataset ranged from 0.66% to 16.98%, while the *R*^2^ of GEMMA-detected tag QTNs ranged from 1.11% to 10.00%. Similar results were observed in the GB and RGAB genotypic datasets (**Table 1**). Of note, eight tag QTNs were identified in both ALL and GB genotypic datasets, and explained 1.06% to 12.72% of the total variation for pasmo severity (**Supplementary Table S3** and **Supplementary Figure S2B**). The position of all tag QTNs for pasmo severity are illustrated on a CIRCOS map (**Figure 4**). A total of eight tag QTNs were considered large-effect QTNs, i.e., *R*^2^ ≥ 10% (**Table 2** and **Supplementary Table S4**). Based on these QTNs, significant negative correlations were observed between the number of favorable alleles (NFAs) in an accession and the six pasmo severity datasets (PAS2012–PAS2016 and PASmean) (*r* = −0.39 ~ −0.71) (**Supplementary Figure S3A–F**), with the strongest correlation observed in the PASmean dataset (*r* = −0.71) (**Supplementary Figure S3F**).

**Table 2:** Large-effect quantitative trait nucleotides (QTNs) and QTN-by-environment interactions (QEIs) detected in two genotypic datasets.

GD	*R^2^* (%)	QTN/QEI	Chr	Pos	Gene ID	Annotation
RGAB	10.79	QTN-Lu4-14335180	4	14335180	*Lus10041466*	TM-CC
RGAB	27.34	QEI-Lu5-1569144	5	1569144	*Lus10004719*	TNL
RGAB	16.77	QTN-Lu5-1715943	5	1715943	*Lus10008486*	RLK
RGAB	13.34	QTN-Lu5-15543693	5	15543693	*Lus10024053*	TM-CC
RGAB	11.88	QEI-Lu5-15543693	5	15543693	*Lus10024053*	TM-CC
RGAB	10.07	QTN-Lu10-11256857	10	11256857	*Lus10032735*	RLK
RGAB	22.42	QTN-Lu10-11656889	10	11656889	*Lus10032759*	NL
RGAB	17.40	QTN-Lu10-11657307	10	11657307	*Lus10032759*	NL
RGAB	15.77	QTN-Lu12-5214501	12	5214501	*Lus10018309*	TN
GB	13.77	QTN-Lu14-2333894	14	2333894	*Lus10025565*	TM-CC

GD, genotypic dataset: Chr, chromosome: Pos, position: TM-CC, transmembrane coiled-coil protein: TNL, TIR-NBS-LRRs: RLK, receptor-like protein kinase: NL, NBS-LRR. GB, gene-based SNPs: RGAB, resistance gene analog (RGA)-based SNPs.

### Candidate genes for pasmo resistance

**Figure 5:**
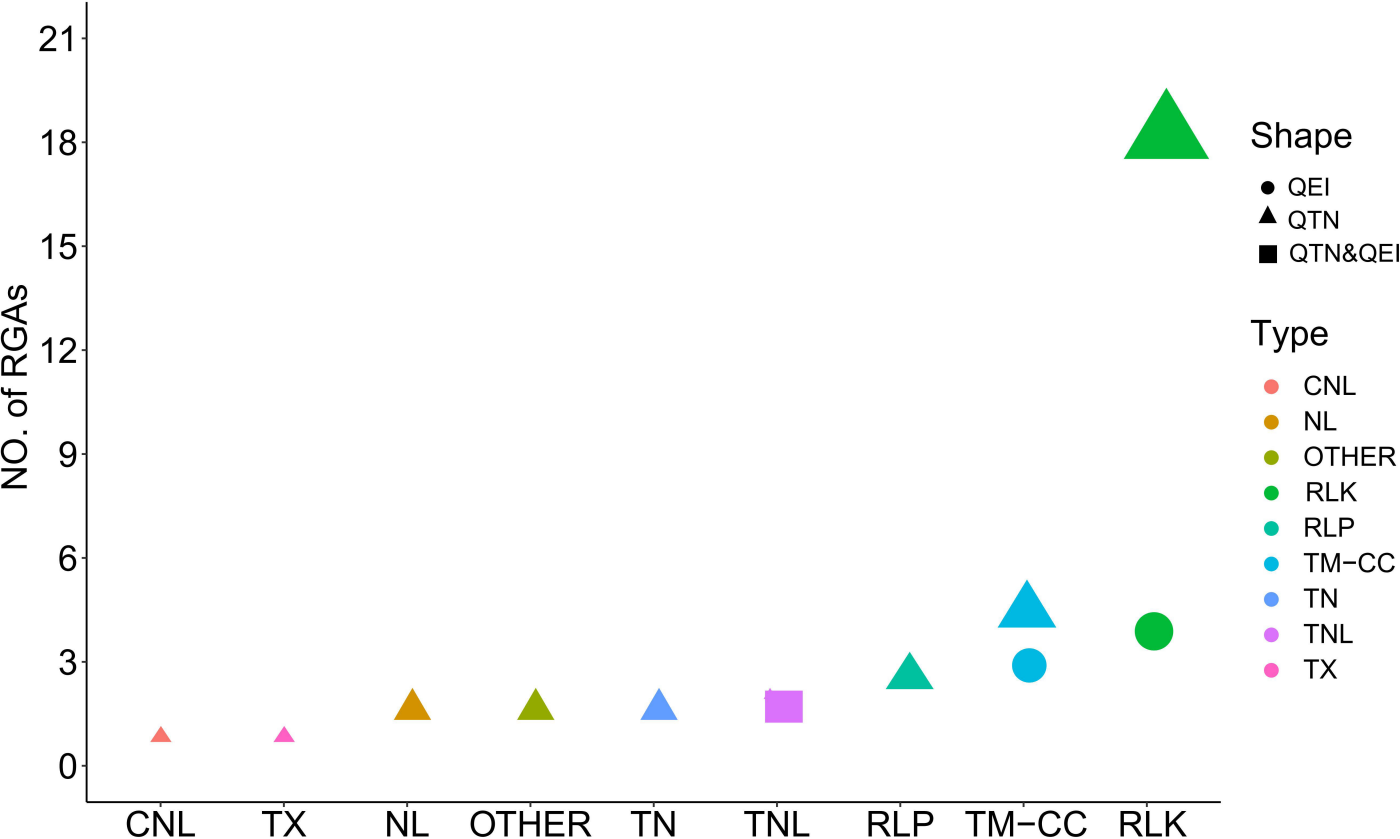
Distribution of candidate resistance gene analogs (RGAs) associated with tag quantitative trait nucleotides (QTNs) and QTN-by-environment interactions (QEIs). RLP, receptor like protein: RLK, receptor like kinase: CNL, CC-NBS-LRR: TNL, TIR-NBS-LRRs: TX, TIR-unknown: NL, NBS-LRR: TN, TIR-NBS: TM-CC,transmembrane-coiled coil protein.

To identify the genes putatively involved in pasmo resistance in flax, we scanned resistance gene analogs (RGAs) within the estimated 4 kb flanking region of the QTNs identified from the ALL genotypic dataset, and identified the tag QTNs located within RGAs as candidate genes for the QTNs identified from the GB or RGAB genotypic dataset. The 37 RGAs that co-localized with 39 tag QTNs were considered candidates for pasmo resistance in flax (**Supplementary Table S4**). These RGAs were mainly classified into eight types, including receptor-like protein (RLP), receptor-like kinase (RLK), TIR-NBS-LRRs (TNL), TIR-unknown (TX), NBS-LRR (NL), TIR-NBS (TN), transmembrane-coiled coil protein (TM-CC), CC-NBS-LRR (CNL), and others. The majority of these RGAs were RLK (19) followed by TM-CC (5) (**Figure 5**).

**Figure 6:**
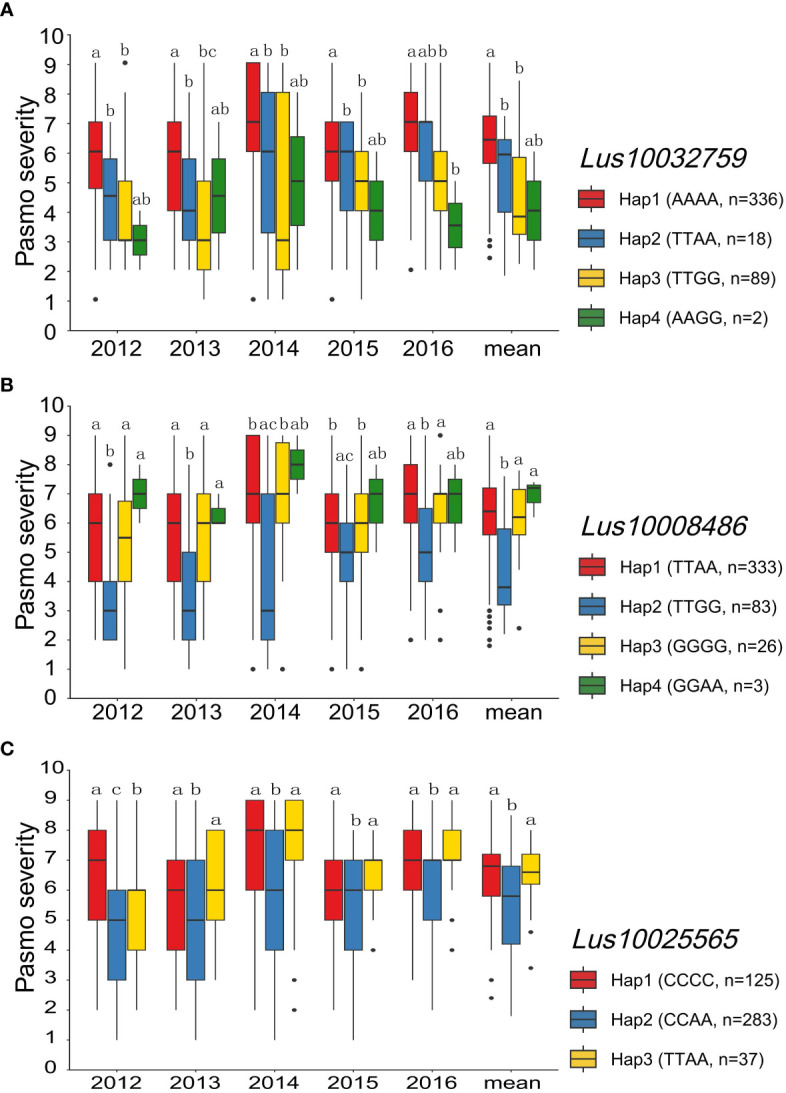
Analyses of the candidate genes *Lus10032759*, *Lus10008486* and *Lus10025565* for pasmo resistance for the five individual years and the mean over years. **(A)** Haplotype and pasmo severity analysis of Lus10032759 in 445 flax accessions. **(B)** Haplotype and pasmo severity analysis of *Lus10008486* in 445 flax accessions. **(C)** Haplotype and pasmo severity analysis of *Lus10025565* in 445 flax accessions. Letters indicate significant differences at the 5% probability level.

Out of the 132 tag QTNs, QTN-Lu10-11656889 was identified by four models from the RGAB genotypic dataset, and explained 22.42% of the total variation. This QTN was located within the *NL* gene *Lus10032759* (**Supplementary Figure S4A** and **Supplementary Table S4**) which had four haplotypes Hap1 (AAAA, n = 336), Hap2 (TTAA, n = 18), Hap3 (TTGG, n = 89), and Hap4 (AAGG, n = 2) (**Figure 6A**). Significant differences in pasmo severity were observed between accessions with the Hap1 and Hap3 in all six phenotypic datasets, with accessions carrying Hap3 exhibiting lower pasmo severity than those carrying Hap1 (**Figure 6A**). QTN-Lu5-1715943 also had a relatively large effect (*R*^2^ = 16.77%) in the RGAB genotypic dataset. The candidate gene for this QTN was the RLK-type RGA *Lus10008486* (**Supplementary Figure S4B** and **Supplementary Table S4**). The accessions with Hap2 (TTGG, n = 83) showed significantly lower pasmo severity than those with Hap1 (TTAA, n = 333), Hap3 (GGGG, n = 26), and Hap4 (GGAA, n = 3), again in almost all six phenotypic datasets (**Figure 6B**) In addition, the TM-CC type RGA *Lus10025565*, identified by the QTN-Lu14-2333894, also had a relatively large effect (*R*^2^ = 13.77%), as detected from the GB genotypic dataset (**Supplementary Figure S4C** and **Supplementary Table S4**). The pasmo severity of accessions with Hap2 (CCAA, n = 283) was significantly different from those with other two haplotypes, with lower pasmo severity observed in Hap2 accessions than in Hap1 (CCCC, n = 125) and Hap3 (TTAA, n = 37) accessions (**Figure 6C**).

### QEI detection and candidate genes

**Figure 7:**
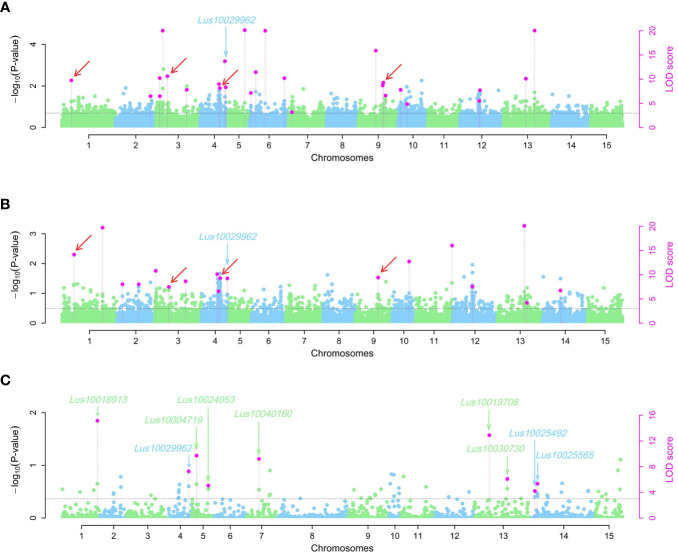
Manhattan plots for pasmo resistance associated QTN-by-environment interactions (QEIs) identified using the 3VmrMLM model for three single nucleotide polymorphisms (SNPs) datasets: ALL **(A)**, GB **(B)**, and RGAB **(C)**. Black horizontal lines in the Manhattan plots represent the genome-wide significant threshold. The red arrows indicate the QEIs co-detected in ALL **(A)** and GB **(B)** SNP datasets. The green and blue arrows indicate the candidate genes detected in ALL, GB, and RGAB SNP datasets. ALL, all SNPs; GB, gene SNPs; RGAB, resistance gene analog (RGA)-based SNPs.

Using the 3VmrMLM model, a total of 50 QEIs underlying pasmo resistance in flax were identified from the ALL, GB, and RGAB genotypic datasets across the five individual year phenotypic datasets (PAS2012–PAS2016), as shown in **Figures 4**, **7A–C**, and **Supplementary Table S5**. Overall, 27, 18, and nine QEIs were identified from the ALL, GB, and RGAB genotypic datasets, respectively. Four of these QEIs were detected in both the ALL and GB genotypic datasets: QEI-Lu1-3346281, QEI-Lu3-4320878, QEI-Lu4-14847340, and QEI-Lu9-17104439. Notably, no QEI loci for pasmo resistance were detected on chromosomes 8 and 15 (**Supplementary Table S5**).

The following four QEIs located on genes and detected from the GB or RGAB dataset were also identified as tag QTNs: QEI-Lu5-15543693 (*R*^2^ = 11.88%), QEI-Lu11-19819154 (*R*^2^ = 5.10%), QEI-Lu14-2333894 (*R*^2^ = 6.01%), and QEI-Lu14-1935665 (*R*^2^ = 2.85%) (**Supplementary Table S2**, **S5** and **Supplementary Figure S5**).

**Figure 8:**
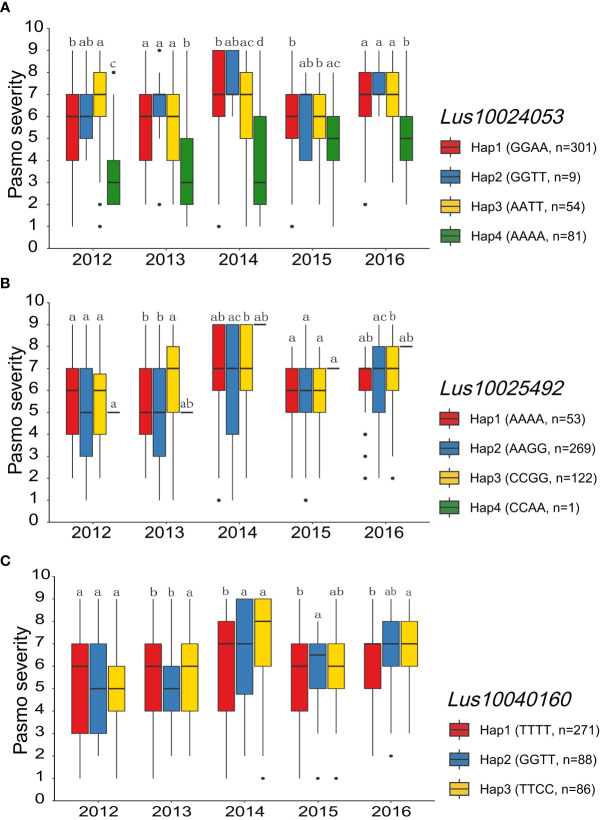
Analyses of the candidate gene *Lus10024053*, *Lus10025492* and *Lus10040160* for pasmo resistance associated QTN-by-environment interactions (QEIs) for the five individual years. **(A)** Box plot of pasmo severity of Lus10024053 haplotypes. **(B)** Box plot of pasmo severity of *Lus10025492* haplotypes. **(C)** Box plot of pasmo severity of *Lus10040160* haplotypes. Letters indicate significant differences at the 5% probability level.

The nine RGAs predicted as candidate genes for ten QEIs were further analyzed (**Supplementary Table S6** and **Figure 5**). The TM-CC type RGA *Lus10024053* was the candidate gene for the large-effect QEI-Lu5-15543693, with Hap1 (GGAA, n = 301), Hap2 (GGTT, n = 9), Hap3 (AATT, n = 54), and Hap4 (AAAA, n = 81). The severity of pasmo infection in accessions with Hap4 was significantly lower than that of accessions with the other three haplotypes in the PAS2012, PAS2013, PAS2014, and PAS2016 datasets (**Figure 8A**; **Supplementary Figure S4D**; **Supplementary Table S6**). Additionally, the RLK type RGA *Lus10025492* was identified as the candidate gene of QEI-Lu14-1935665, with Hap1 (AAAA, n = 53), Hap2 (AAGG, n = 269), Hap3 (CCGG, n = 122), and Hap4 (CCAA, n = 1). A significantly lower pasmo severity of Hap2 was observed in PAS2013, PAS2014, and PAS2016 compared to Hap3 (**Figure 8B**; **Supplementary Figure S4E**; **Supplementary Table S6**). Similarly, the RLK RGA *Lus10040160* was identified as the candidate gene of QEI-Lu7-4573781. *Lus10040160* hasHap1 (TTTT, n = 271), Hap2 (GGTT, n = 88), and Hap3 (TTCC, n = 86), and significant differences in pasmo severity were observed between the Hap1 and Hap3 in the PAS2013, PAS2014, and PAS2016 datasets. The pasmo resistance level of accessions with Hap3 was significantly higher than that of accessions with Hap1 in those years (**Figure 8C**; **Supplementary Figure S4F**; **Supplementary Table S6**).

## Discussion

### Comparison across GWAS models

The detection of QTNs in GWAS can vary depending on the statistical algorithms implemented in the models. In this study, three genotypic datasets (ALL, GB, and RGAB) were evaluated across six phenotypic datasets for pasmo resistance. The results showed that the 3VmrMLM model detected the most QTNs, followed by mrMLM and GEMMA. Most of the QTNs detected by at least two models were identified by 3VmrMLM. These findings support previous studies indicating that multi-locus models outperform single-locus models in QTN detection, and suggest that 3VmrMLM high statistical power and low false positive rate are advantageous (Cui et al., 2018; Hou et al., 2018; Zhong et al., 2021; He et al., 2022; Li et al., 2022b; Liu et al., 2022; Yu et al., 2022; Zhang et al., 2022).

After removing non-significant QTNs, the most tag QTNs were also identified by 3VmrMLM, followed by mrMLM and FarmCPU. The largest *R*^2^ ranges were also observed in 3VmrMLM identified tag QTNs in all four models used, indicating its ability to identify tag QTNs with either large or small effects. Taken together, the 3VmrMLM model seems a good alternative to other single-locus and multi-locus models in GWAS. The 3VmrMLM model was developed to effectively detect main-effect QTNs, QEIs, and QQIs while providing unbiased estimates of their effects through an analysis of variance (ANOVA) model. This model builds upon the framework of compressed variance component mixed model (Li et al., 2022a) and presents technical improvements. One key reason for the superior performance of the 3VmrMLM model is its ability to consider all genetic effects in the mixed genetic model while simultaneously controlling for all polygenic backgrounds (Li et al., 2022a; Li et al., 2022b).

### Evaluation of QTNs associated with pasmo resistance

Flax pasmo resistance is a quantitative trait, characterized by features of quantitative genetics. The challenge of visually measuring the resistance prompted us to adopt the pasmo severity scale (0–9) as a means to assess the severity of pasmo disease symptoms in our experimental genotypes. This severity scale provides a practical and standardized approach for quantitatively representing pasmo disease symptoms, despite its categorical appearance in scoring pasmo resistance. By utilizing this scale, we were able to capture the gradation in the expression of the trait among different genotypes, enabling a more comprehensive evaluation of the potential genetic factors influencing pasmo severity. Notably, this method has been commonly used for evaluating powdery mildew resistance in flax (You et al., 2022).

Using the multiple years’ flax pasmo severity data, a total of the 132 tag QTNs were detected in this study, out of which 29 were previously reported in a study of the flax core collection consisting of 370 accessions that utilized the same phenotyping method (He et al., 2018). In the aforementioned study, which focused on the 370 flax accessions, a subset of the current study, a total of 67 QTLs with large effects were identified by GWAS using various models, including GLM, MLM, FarmCPU, GEMMA, mrMLM, FASTmrEMMA, ISIS EM-BLASSO, pLARmEB, pKWmEB and FASTmrMLM models (He et al., 2018). Furthermore, four tag QTNs (QTN-Lu8-17271798, QTN-Lu13-2007925, QTN-Lu15-974597, and QTN-Lu13-14282050) were found to be situated within 1.01–16.97 kb upstream/downstream of QTLs previously reported in He et al. (2018) (**Supplementary Table S3**). To identify novel QTNs and their corresponding candidate genes associated with pasmo resistance in flax, multi-model and multi-environment GWAS were conducted using the ALL, GB, and RGAB genotypic datasets. A total of 31 (ALL), 49 (GB), and 27 (RGAB) novel tag QTNs were identified using 445 flax accessions (370 core accessions and 75 SBLs), which is an improvement compared to our previous study. Eight tag QTNs (*R*^2^ = 1.11%–12.72%) were identified in both the ALL and GB datasets. Additionally, one and seven out of eight large-effect QTNs (*R*^2^ ≥ 10.00%) were identified from the GB and RGAB datasets respectively (**Table 2** and **Supplementary Table S3**). Among the tag QTNs with the top five *R*^2^ (16.98%–22.42%), two, two and one tag QTNs were identified from the GB, RGAB, and ALL datasets, respectively (**Supplementary Table S3**). These results are consistent with previous studies suggesting that using gene-based or RGA-based SNPs for GWAS is beneficial for detecting QTNs with large effects and predicting key candidate genes (Huang et al., 2011; Zhu et al., 2018; Deng et al., 2020; You et al., 2022; Zhang et al., 2022). Therefore, the use of gene-based or RGA-based SNPs for GWAS is a powerful and efficient approach for identifying QTNs with large and small effects.

### Candidate genes associated with pasmo resistance and their effects on main-effect QTNs and QEIs

Main-effect QTNs are QTNs with stable effects across different environments, while QEIs represent loci that may be effective only in some environments. Given the needs of global climate change and phenotypic plasticity research, QEIs have the potential to be exploited to dissect complex traits in future GWAS. In this study, candidate gene prediction of QTNs and QEIs was based on well-characterized RGAs in flax. RGAs have been identified as key candidate genes underlying plant disease resistance in several studies (Kassa et al., 2017; He et al., 2018; Fu et al., 2020; You et al., 2022). A total of 37 RGAs were identified as potential candidate genes of 39 tag QTNs and nine as candidates for ten QEIs. They were summarized into RLK, TM-CC, and NBS-LRR type RGAs. In general, the *RLK*, *TM-CC*, and *NBS-LRR* genes account for a large proportion of *R* genes, playing important roles in plant disease resistance against fungal pathogens. Well-known examples include wheat leaf rust resistance conferred by the *Lr21* (*NBS-LRR*) gene (Huang et al., 2003), resistance to the hemi-biotrophic fungus *Phytophthora infestans* conferred by the potato *R7* (*NBS-LRR*) gene (Leister et al., 1996; Hammond-Kosack and Jones, 1997), broad-spectrum mildew resistance conferred by the Arabidopsis *RPW8* (*TM-CC*) gene (Xiao et al., 2001), and rice blast resistance conferred by the *Pi-d2* (*RLK*) gene (Chen et al., 2006). The RLK, TM-CC, and NBS-LRR type RGAs associated with pasmo resistance in this study may contribute to a better understanding of the genetic mechanisms underlying pasmo resistance in flax. Furthermore, the molecular mechanisms of these candidate genes warrant further validation.

### Breeding applications of pasmo resistance associated QTNs

The present study revealed significant differences in pasmo resistance levels between linseed, fibre accessions, and SBLs within a flax genetic panel. Interestingly, 75 SBLs exhibited higher pasmo resistance levels than the flax core collection, which included 370 accessions (**Figure 1C**). Moreover, the number of favorable alleles (NFA) in fibre accessions was greater than in linseed accessions, and fibre accessions with more favorable alleles were found to be more resistant to pasmo than linseed accessions (**Supplementary Figure S6**), as demonstrated in a previous study (He et al., 2018). Flax have obtained commercial importance due to the utilization of the stem for high quality fiber (Oomah, 2001; You et al., 2019; Rahman and Hoque, 2023). One of the major objectives in the fiber flax breeding program is to improve fiber yield and quality (Galinousky et al., 2020; Rahman and Hoque, 2023). The productivity of fiber flax is severely affected by devastating fungal disease pasmo, which causes yield loss and fiber quality reduction (Yadav et al., 2022). Therefore, the 75 SBLs represent valuable genetic resources for improving pasmo resistance in elite varieties through direct hybridization.

Negative correlations were observed between the NFA and pasmo resistance of the five-year pasmo severity (PAS2012–PAS2016) and PASmean datasets in **Supplementary Figure S3A–F** (*r* = −0.39 ~ −0.71), with the highest correlation found in the PASmean dataset (*r* = −0.71). This additive effect of identified tag QTNs suggests that accessions carrying more favorable alleles are suitable for high pasmo resistance breeding through the pyramiding of loci. For example, SBL 8031 had 17 favorable alleles (PASmean = 2.2), SBL 8040 had 17 favorable alleles (PASmean = 2.4), and SBL 8032 had 18 favorable alleles (PASmean = 2.4).

Although large-effect tag QTNs, such as QTN-Lu10-11656889 (*R*^2^ = 22.42%) and QTN-Lu12-2992110 (*R*^2^ = 16.68%), may be available for improving pasmo resistance through marker-assisted selection (MAS), several tag QTNs with small effects would be better captured through genomic prediction/selection with the aim to transform flax breeding from a slow and labor-intensive mode into an efficient and accurate one. The breeding values of complex traits, such as pasmo resistance, are predicted by cross-validated models, which are an alternative strategy to MAS (Lipka et al., 2015; Poland and Rutkoski, 2016; He et al., 2019; You et al., 2022). Marker-assisted backcrossing and genomic selection/prediction strategies have already significantly enhanced disease resistance in many crops (Buerstmayr et al., 2008; Buerstmayr et al., 2009; Poland and Rutkoski, 2016; Crossa et al., 2017; He et al., 2019; Xu et al., 2021).

The QEI loci identified in this study constitute an alternative genetic information for improving flax pasmo disease, specifically to cope with environmental changes. These QEI loci can be useful for predicting the performance of flax varieties in specific environments. By identifying specific genetic markers associated with QEI loci, breeders can develop flax varieties that are better adapted to specific environmental conditions. The combined utilization of pasmo resistance-associated QTNs and QEIs holds the promise of driving the molecular breeding of flax with broad-spectrum and durable resistance against *Septoria linicola*.

## Conclusion

Our study demonstrates that pasmo resistance in flax is a complex trait, controlled by multiple genes, and influenced by gene-environment interactions. The 3VmrMLM model, which detected more QTNs and QEIs, is a promising alternative to other multi-locus GWAS models. Gene-based and RGA-based SNPs as genotypic datasets in GWAS proved to be efficient for identifying QTNs with both large and small effects and predicting candidate genes. Our research identified 372 significant QTNs and 50 QEIs, providing potential targets for improving pasmo resistance in flax breeding programs. Furthermore, we identified 37 RGAs for 39 tag QTNs and nine RGAs for ten QEIs, suggesting the potential involvement of RLK, TM-CC, and NBS-LRR genes in pasmo resistance. Our findings on gene–environment interactions can guide breeding strategies that account for environmental factors. The 50 QEI loci identified in our study can help improve our understanding of the genetic mechanisms involved in pasmo resistance and its interactions with environmental factors, ultimately leading to the development of more resilient and better adapted flax varieties. Our study has important implications for the sustainable production of flax and provides valuable information for developing improved flax varieties with enhanced pasmo resistance, which is critical for ensuring the long-term viability of this important oil and fiber crop. The large-effect QTNs and candidate genes identified in this study can be used as molecular markers for marker-assisted selection in future studies to accelerate the breeding process for pasmo-resistant flax varieties.

## Data availability statement

The raw sequence data for flax core collection presented in this study are deposited in the NCBI repository (project number: PRJNA707038).

## Author contributions

FY, SC and LH conceived and designed this research project. KR produced the breeding lines and provided all phenotypic data. LH, YS, YC and HW undertook the analysis of all available data. LH and YS contributed to the writing of the original draft. FY, SC, and LH discussed the results, guided the entire study, participated in data analysis, and revised the manuscript. All authors contributed to the article and approved the submitted version.
