# Supplementary material for: Genome-wide association studies using multi-models and multi-SNP datasets provide new insights into pasmo resistance in flax
Source: Front Plant Sci. 2023 Oct 25;14:1229457. doi: 10.3389/fpls.2023.1229457 (PMC10634603; doi:10.3389/fpls.2023.1229457)
Supplement: Supplementary file 1 [file DataSheet_1.docx]

**Supplementary Figures**


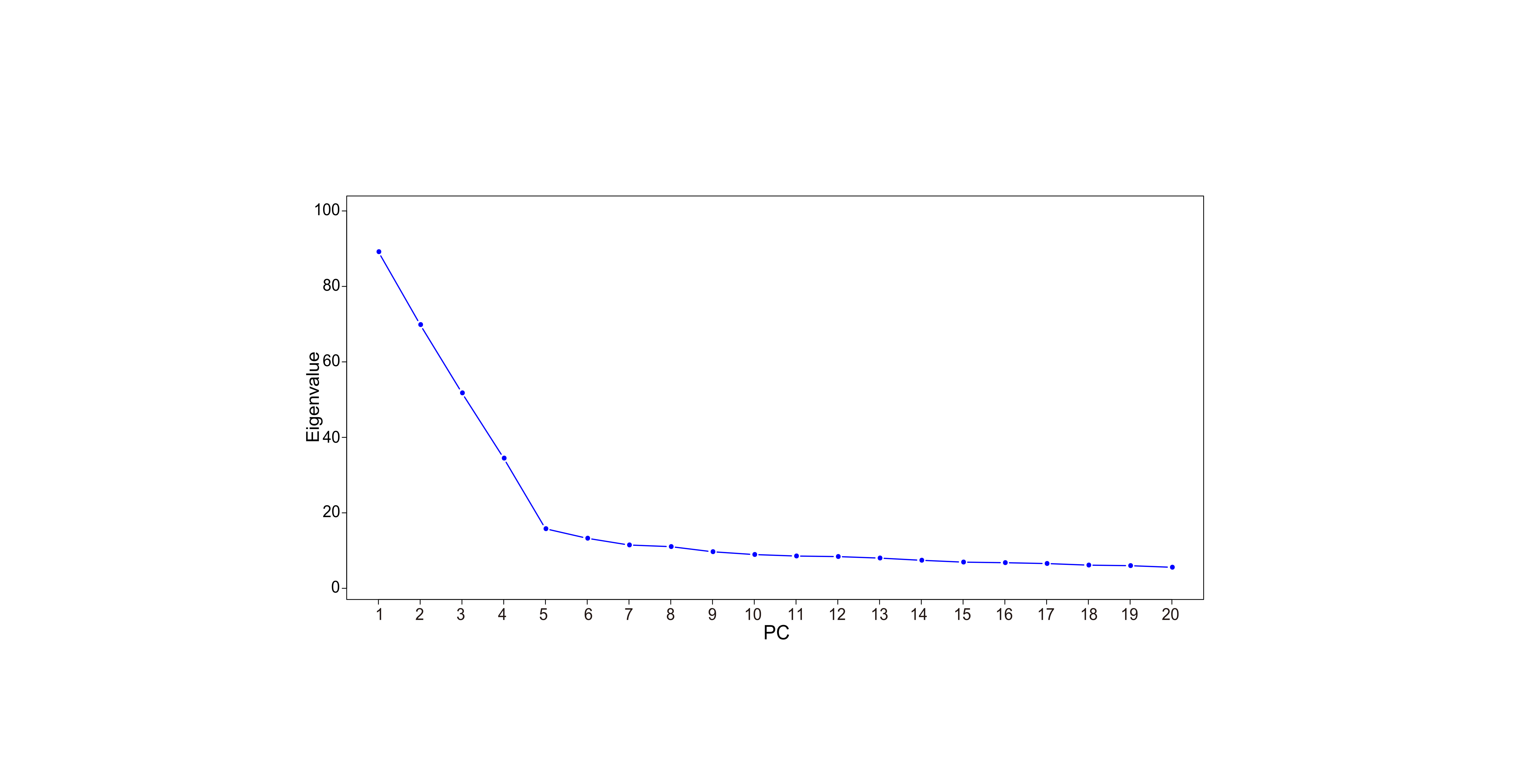


**Supplementary Figure 1:** Plot of eigenvalues calculated for the first 20 principal components (PCs).


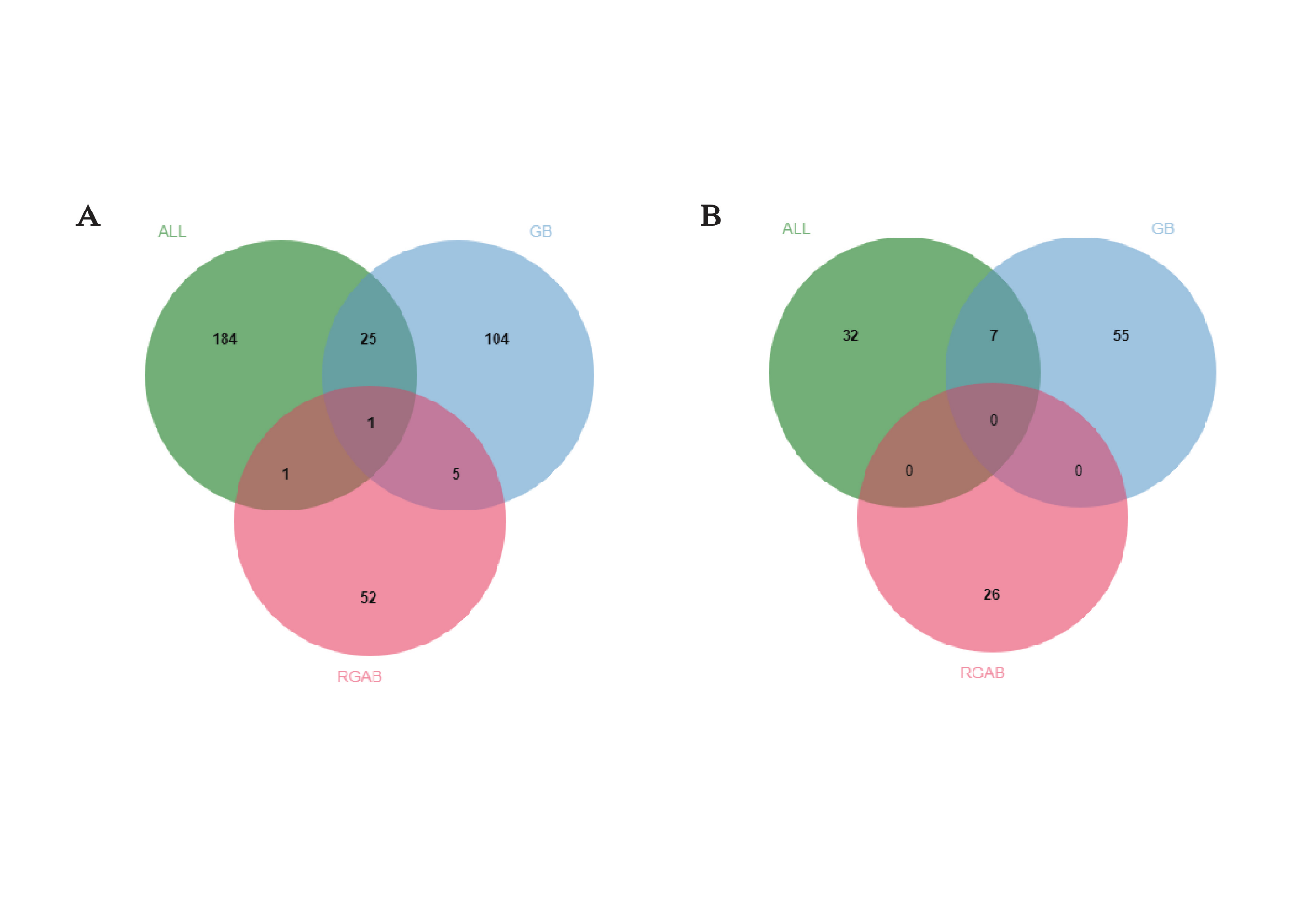


**Supplementary Figure 2:** Venn diagrams of all quantitative trait nucleotides (QTNs) (A) identified for the three genotypic datasets, ALL, GB and RGAB with six phenotypic datasets (2012, 2013, 2014, 2015, 2016, mean) and tag QTNs (B) detected for the three genotypic datasets, ALL, GB and RGAB with five phenotypic datasets (2012, 2013, 2014, 2015 and 2016). ALL: all SNPs; GB: gene-based SNPs; RGAB: resistance gene analog (RGA)-based SNPs.

**
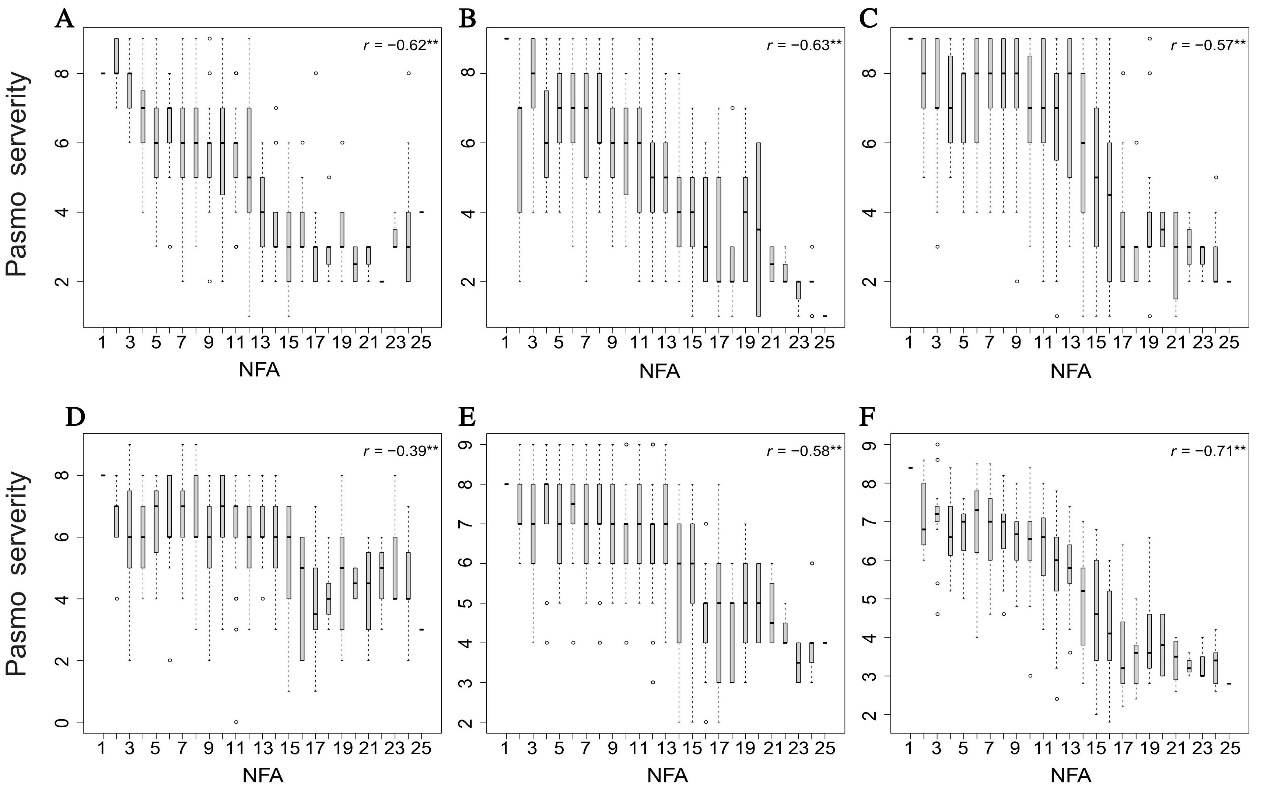
**

**Supplementary Figure 3:** Box plots illustrating the relationship between the number of favorable alleles (NFA) and pasmo severity for the six phenotypic datasets: PAS2012-PAS2016 and PASmean datasets (A-F). ** indicates statistical significance at the 1% probability level.

**
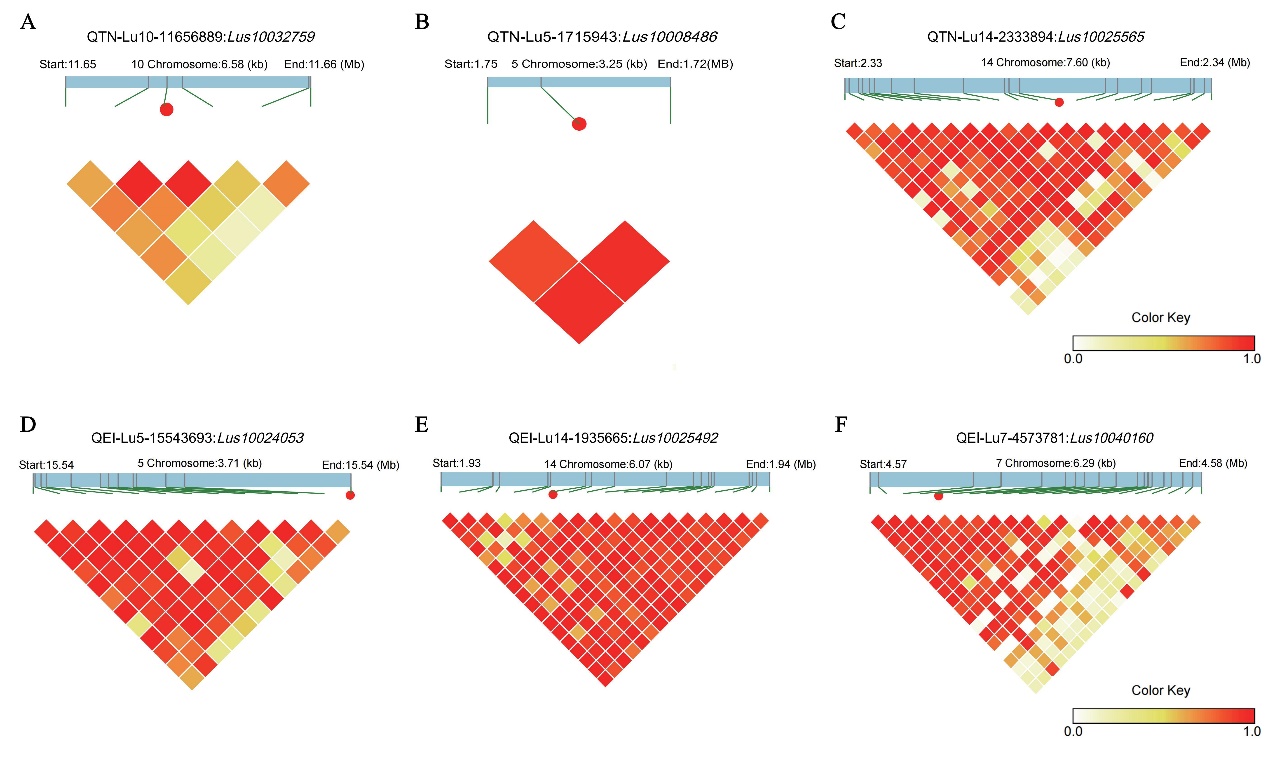
**

**Supplementary Figure 4:** Local linkage disequilibrium block analyses of pasmo resistance associated QTNs, QEIs, and their corresponding candidate genes. Three QTNs (A-C) and three QEIs (D-F). The red circle indicates tag QTN or QEI locus.


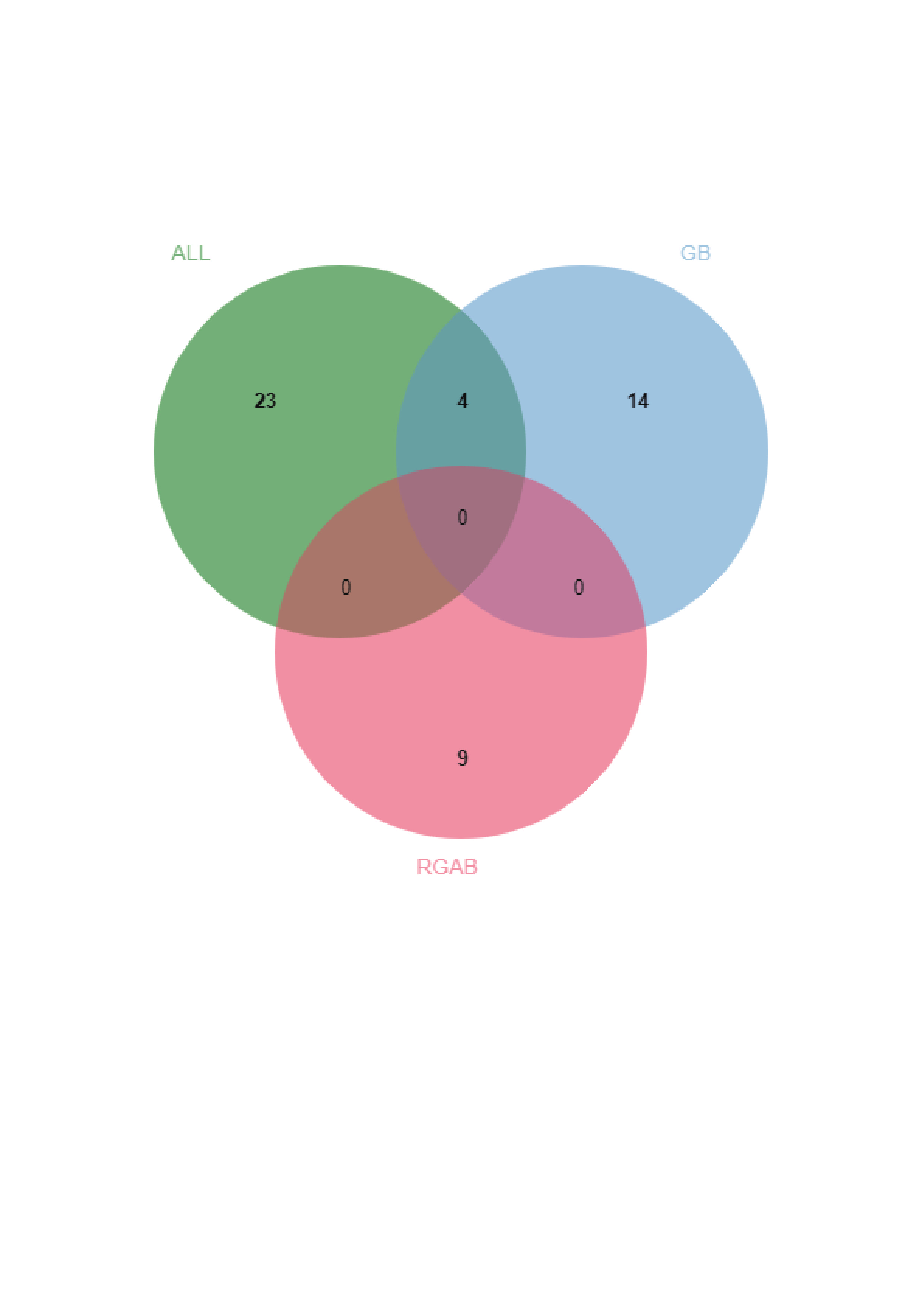


**Supplementary Figure 5:** Venn diagram of QTN-by-environment interactions (QEIs) detected from five phenotypic datasets (2012, 2013, 2014, 2015 and 2016).
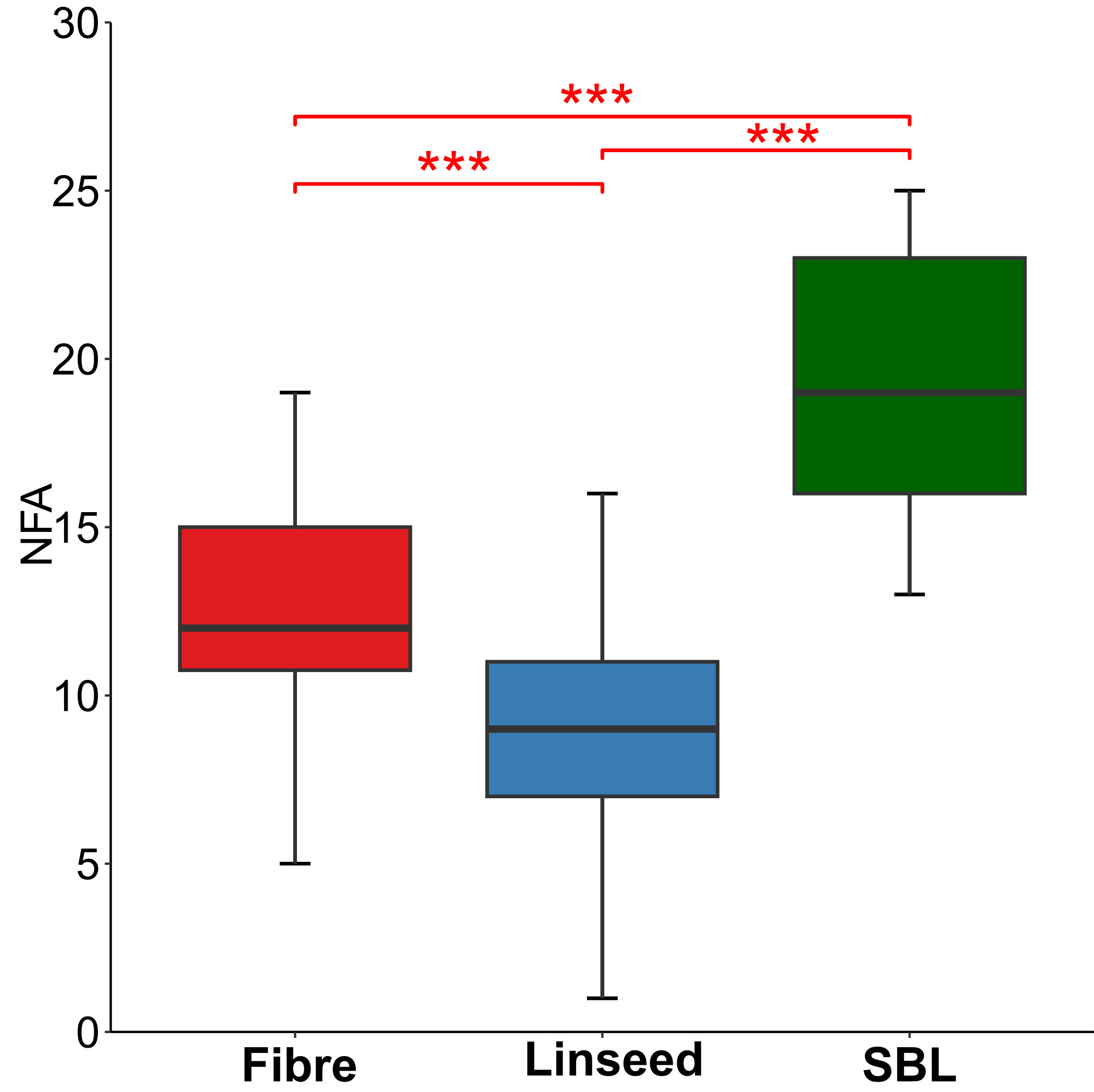


**Supplementary Figure 6:** Relationships between flax morphotypes and the number of favorable alleles (NFAs). *** indicates statistical significance at the 0.1% probability level. SBL: selected breeding line. All SBLs are of linseed morphotype.
